# Transmitter and ion channel profiles of saccadic omnipause neurons and cholinergic non-omnipause neurons in human nucleus raphe interpositus

**DOI:** 10.3389/fnana.2025.1670220

**Published:** 2025-12-01

**Authors:** Ümit S. Mayadali, Maximilian John, Michael Abspacher, Christoph Schmitz, Aasef G. Shaikh, Anja K. E. Horn

**Affiliations:** 1 Chair of Vegetative Anatomy, Institute of Anatomy, Faculty of Medicine, LMU Munich, Munich, Germany; 2 Chair of Neuroanatomy, Institute of Anatomy, Faculty of Medicine, LMU Munich, Munich, Germany; 3 Department of Neurology, University Hospitals, Cleveland VA Medical Center, Case Western Reserve University, Cleveland, OH, United States

**Keywords:** high-voltage-gated potassium channels, low-voltage activated calcium channels, glutamate, GABA, Glycine, perineuronal nets

## Abstract

**Background:**

Omnipause neurons (OPN) are glycinergic neurons that tonically inhibit burst neurons between saccades. In primates, OPNs are located bilaterally around the midline at the level of the traversing rootlets of the abducens nerve in the pontine brainstem forming the nucleus raphe interpositus (RIP). Healthy OPNs are previously characterized by dense perineuronal net (PN) ensheathment, parvalbumin (PAV) and voltage-gated potassium channel Kv1.1 and Kv3.1 expression.

**Motivation:**

The ion channel and transmitter profile of OPNs in human has not been established. The further characterization of OPNs should allow for local delineation of OPNs from other types of neurons found in RIP, as well as identifying potential markers for eye movement disorders such as opsoclonus myoclonus syndrome.

**Methods:**

Double immunoperoxidase based-stainings of transverse pontine sections containing human RIP for aggrecan (ACAN) and non-phosphorylated neurofilaments (SMI32) was used to identify OPNs. In consecutive thin paraffin sections, stainings using antibodies against low voltage-activated ion channels (HCN, Cav3) and transmitter related proteins were performed.

**Results:**

A separate but morphologically similar population to OPNs was identified around the midline at the same level as OPNs in human pontine sections. This population was cholinergic, lacked PNs, but was labeled by SMI32. Further examination revealed that OPNs and cholinergic non-OPN populations differ in their ion channel (Kv3.1, HCN1-2, Cav3.2) and transmitter related protein (GABRA, GAD, GlyR, vGlut, GluR) expression.

**Conclusion:**

OPNs and cholinergic non-OPNs are located intermingled within the traditionally identified RIP, however they expressed distinct histochemical signatures from OPNs. Although the functional significance of the cholinergic non-OPN population in human brainstem is unclear, these findings suggest important distinguishing features that could be missed in histopathological examinations of post-mortem cases with saccadic disorders.

## Introduction

### Physiological substrates of fast eye movements

With our body at rest or in motion, we are constantly exploring our environment with fast scanning eye movements that “jump” between fixation targets, therefore are being called saccades. To avoid impairment of proper vision especially during own body movements saccadic eye movements must be very fast. In human, saccades reach peak velocities up to 400°/sec (Lappe-Osthege et al., 2010) often shown in plots describing the relation peak velocity and amplitude called the “main sequence” (Leigh and Zee, 2015). Recording in awake monkeys revealed the characteristic burst-tonic firing pattern of motoneurons of agonist eye muscles during a saccade. The burst provides the muscle force to overcome the orbital viscous drag, whereas the tonic discharge conveys the new eye position (Fuchs and Luschei, 1970; Robinson, 1970).

The initiation of saccades is generated by the interplay of two neuron types in the brainstem: burst (BN) and omnipause neurons (OPN). Excitatory burst neurons for horizontal saccades lie in the paramedian pontine reticular formation (PPRF), those for vertical saccades in the rostral interstitial nucleus of the medial longitudinal fascicle (RIMLF) in the mesencephalic reticular formation (Horn, 2006). Burst-neurons exhibit a short high-frequent burst of activity prior to and during a saccade, but are otherwise tonically inhibited by OPNs. Only after inhibition of the OPNs by hypothetical local latch neurons, burst neurons are released from inhibition and can fire and generate saccades (Leigh and Zee, 2015). The well-timed and precise interactions of OPNs and BNs (and possibly latch neurons) are essential for the execution of normal saccades. As observed in some neurodegenerative or autoimmune diseases or after vascular or neoplastic lesion, the dysfunction in the premotor saccadic network causes multiple eye movement disorders (e.g., Averbuch-Heller et al., 1995; Thurtell et al., 2007; Rucker et al., 2021), which are not necessarily correlated with neuronal loss of OPNs or BNs (Ridley et al., 1987; Büttner-Ennever et al., 2008). Since the firing characteristics and overall excitability of neurons are determined by their transmitter and ion channel profiles (Catterall, 1984), efforts have been made to identify these profiles in identified cell groups of the oculomotor and vestibular system in different species (Kodama et al., 2020; Mayadali et al., 2022; Mayadali et al., 2024). However, critical information and insight that govern the firing characteristics of OPNs and BNs in human is still missing.

### Omnipause neurons, function and histochemical characteristics

Saccadic omnipause neurons (OPNs), also known as omnidirectional pause neurons, play a crucial role in the control of saccadic eye movements, which are rapid, simultaneous movements of both eyes that shift the line of sight from one point to another. OPNs are unique in that they exhibit continuous firing during periods of visual fixation and pause their activity just before and during saccadic movements (Cohen and Henn, 1972; Luschei and Fuchs, 1972; Keller, 1974). This pausing mechanism is essential for the initiation and proper execution of saccades, as it disinhibits the burst neurons that generate the high-velocity eye movements (Shinoda et al., 2008). Both in humans and monkeys, OPNs contribute to the precision and accuracy of saccades by ensuring that these rapid eye movements are well-timed and coordinated (Shaikh et al., 2007; Shaikh et al., 2008). Experimental lesions of OPNs result in reduction of saccade peak velocity, but not in saccade accuracy (Kaneko, 1996; Soetedjo et al., 2002).

In monkey, the OPNs form a distinct cytoarchitectonic entity consisting of a cell column on either side of the midline, which are distinct from neighboring raphe nuclei, thus were termed nucleus raphe interpositus (RIP) (Büttner-Ennever et al., 1988). In all species, where identified, RIP is located at levels where the dorsoventrally traversing fibers of the abducens nerve are seen (rat: Hittinger and Horn, 2012; cat: Strassman et al., 1987; monkey: Büttner-Ennever et al., 1988). The OPNs show a characteristic morphology as medium-sized neurons with long horizontally oriented dendrites reaching far into the adjacent reticular formation and some crossing the midline (Strassman et al., 1987). Based on location, morphology and histochemical properties, RIP has been identified in the human brain stem as well (Büttner-Ennever et al., 1988; Horn et al., 2003a). In contrast to rhesus monkey, the human RIP forms a less compact cell group with less densely packed cell columns around the midline (Büttner-Ennever and Horn, 2014).

### Biophysiological and histochemical characteristics of omnipause neurons

OPNs discharge at a high tonic rate of up to 150 Hz in cat (Evinger et al., 1982; McCrea et al., 1987; Strassman et al., 1987; Everling et al., 1998) and up to 200 Hz in monkey (Henn et al., 1984). This neuronal activity goes along with a high cytochrome oxidase activity (Büttner-Ennever et al., 1988), expression of the calcium-binding protein parvalbumin and a condensed extracellular matrix formation referred to as perineuronal nets (PN) usually associated with highly active neurons (Härtig et al., 1999; Horn et al., 2003a). Only recently, fast voltage-gated potassium channels Kv1.1 and Kv3.b have been identified in OPNs in monkey and human (Mayadali et al., 2019). Different independent anatomical methods in monkey revealed glycine as the neurotransmitter of OPNs (Horn et al., 1994). In addition, monkey OPNs receive strong input from glutamatergic, GABAergic and glycinergic terminals (Horn et al., 1994), the latter most probably conveying the pause during saccades (Kanda et al., 2007).

The development of neuromimetic mathematical models, which are based on experimental data, take these properties of OPNs in account. Accordingly, hypotheses regarding the cause of symptoms in ocular flutter or oscillations, as seen in opsoclonus, suggest alterations in the physiology of OPNs and/or BNs rather than structural damage alone (Ridley et al., 1987; Ramat et al., 2007; Shaikh et al., 2008). For example, simulation studies of saccades generated with a conductance-based mathematical model involving post-inhibitory rebound (PIR) (Enderle and Engelken, 1995) could demonstrate that inclusion of *t*-current mediated by slow-voltage gated calcium-channels of Cav3 family can cause a slowing of saccades after OPN lesion as found in animal experiments (Kaneko, 1996; Soetedjo et al., 2002; Miura and Optican, 2006).

To build a bridge between histochemistry and firing characteristics of neurons in the oculomotor system, we investigated the ion channel and neurotransmitter profiles of OPNs in human. The findings will serve to establish a baseline for further studies on clinical post-mortem cases with saccadic disorders, thereby enabling the evaluation of the hypothesis that states neurophysiological changes as etiology for oscillations.

For a targeted investigation of only the OPNs in humans, the neurons within the less sharply circumscribed RIP were systematically characterized histochemically in order to identify and exclude neurons with other properties from the analysis.

## Materials and methods

Brains extracted from 3 human cases with post-mortem delays up to 27 h were fixed in 4% paraformaldehyde solution in 0.1 M phosphate buffer (pH 7.4) for between 2–7 days. The age of the donors ranged from 57 years to 80 years, with no known history of neurological disease and included 1 male and 2 female cases. The brainstems were cut into 1–2 cm thick tissue blocks, which were then embedded in paraffin for sectioning in the transverse plane. 5–10 μm thick paraffin sections were then processed with peroxidase-based immunohistochemistry methods identical to previously described protocol in monkey abducens and trochlear nuclei (Mayadali et al., 2024). Information on primary antibodies specifically used in this study are summarized in Table 1.

**Table 1:** Information about host species, sources and dilutions of primary antibodies used in this study.

Antibody	Host	Antigen	Manufacturer	Antibody registry number (RRID)	IHC dilution
ACAN	Mouse/Monoclonal	Aggrecan	Acris Antibodies GmbH, 32052 Herford, GERMANY	AB_972582	1:75
ChAT	Goat/Polyclonal	Choline Acetyltransferase	Chemicon, Temecula, CA, USA	AB_2079751	1:50
Kv1.1	Rabbit/Polyclonal	Voltage-Gated Potassium Channel 1.1	Alomone Labs Jerusalem BioPark (JBP)	AB_2040144	1:500
Kv3.1b	Rabbit/Polyclonal	Voltage-Gated Potassium Channel 3.1b	(Weiser et al., 1995)	Härtig (AB_2040166)	1:6000
GABRA1	Rabbit/Polyclonal	Gaba receptor alpha 1	Proteintech, Planegg/Martinsried, GERMANY	AB_2108692	1:250
GAD	Rabbit/Polyclonal	Glutamate Decarboxylase 65 & 67	Chemicon, Temecula, CA, USA	AB_90715	1:2000
vGlut1	Rabbit/Polyclonal	Vesicular Glutamate Transporter 1	Synaptic Systems, Göttingen, GERMANY	AB_887875	1:3000
vGlut2	Mouse/Monoclonal	Vesicular Glutamate Transporter 2	Chemicon, Temecula, CA, USA	AB_2187552	1:4000
SMI32	Mouse/Monoclonal	Nonphosphorylated Neurofilament marker H	SM1353, Acris Antibodies	AB_2715852	1:2500
GluR2/3	Rabbit/Polyclonal	Glutamate (AMPA) Receptor 2/3	Chemicon, Temecula, CA, USA	AB_90710	1:500
Cav3.1	Rabbit/Polyclonal	T-Type Voltage-Gated Calcium Channel 3.1	Alomone Labs Jerusalem BioPark (JBP)	AB_2039779	1:1000
Cav3.2	Rabbit/Polyclonal	T-Type Voltage-Gated Calcium Channel 3.2	Alomone Labs Jerusalem BioPark (JBP)	AB_2039781	1:1000
Cav3.3	Rabbit/Polyclonal	T-Type Voltage-Gated Calcium Channel 3.3	Alomone Labs Jerusalem BioPark (JBP)	AB_2039783	1:1000
HCN1	Rabbit/Polyclonal	Hyperpolarization-activated cyclic nucleotide-gated channel 1	Thermo Fischer Scientific, MA, USA	AB_2735891	1:400
HCN2	Rabbit/Polyclonal	Hyperpolarization-activated cyclic nucleotide-gated channel 2	Thermo Fischer Scientific, MA, USA	AB_2735892	1:1500
HCN4	Guinea Pig/Polyclonal	Hyperpolarization-activated cyclic nucleotide-gated channel 4	Alomone Labs Jerusalem BioPark (JBP)	AB_2340957	1:200
GlyR1α	Mouse/Monoclonal	Glycine Receptor 1α	Synaptic Systems, Göttingen, GERMANY	AB_887722	1:300
CSPG	Mouse/Monoclonal	Chondroitin Sulfate Proteoglycan	Chemicon, Temecula, CA, USA	AB_2219944	1:500

### Visualization and tissue analysis

Immunostained paraffin sections of human containing RIP were imaged using a slide scanner (Mirax MIDI, Zeiss) equipped with a plan apochromatic objective (x20). The digitized images were viewed and captured on a computer with the free software Slide Viewer (3DHistech, version 2.6). Corresponding detailed views of equally arranged and magnified images of neighboring sections were analyzed on the computer screen. The same neurons were identified by their location with the help of anatomical landmarks, such as blood vessels. For initial plotting of the analyzed cell types (OPNs and non-OPNs), the counter/marker tool of Slide Viewer was used on images of ChAT/ACAN stainings. Only neurons with visible cell nucleus within a zone of 500 μm from the lateral margins of the midline were evaluated. Taken these labelled images as template, final plots were created using drawing software (Coreldraw 11.0; COREL), also used for arrangement and labeling of the figure plates.

### Description of measurements and statistical analysis

In three human cases, the mean diameter [(d_max_ + d_min_)/2] of OPNs and cholinergic non-OPNs were counted and measured for 566 neurons in total of 28 sections (case H1: *n* = 163 + 82, case H2: *n* = 182 + 66, case H3: *n* = 50 + 23, respectively) using ImageJ software. Membranes of selected neurons with visible nuclei within the designated zone were marked by using the freehand tool, then the parameters such as surface area, circumference, d_max_ and d_min_ were measured by the same software. The measurements were imported into Microsoft Excel for the analysis of mean diameters of OPNs and non-OPNs, standard deviations and finally, the implementation of graphs.

As the next step, statistical significance of the differences observed in mean diameters for OPNs and cholinergic non-OPNs was examined within each case. For the application of two-tailed independent student’s *t*-test for independent means, multiple tools were utilized (MS Excel and free online calculators).

### Antisera

#### Choline acetyltransferase (ChAT)

Cholinergic neurons were detected with an affinity-purified polyclonal goat anti-ChAT antibody (Cat #: AB144P; RRID: AB_2079751; Chemicon, Temecula, CA, USA) directed against the whole enzyme isolated from human placenta, which is identical to the enzyme expressed in the brain (Bruce et al., 1985). This antibody recognizes a 68–70 kDa protein. The appearance and distribution of ChAT-immunopositive neurons identified with this antibody in the present study complies with the respective results of previous studies using paraffin sections (Horn et al., 2018). A 1:50 dilution was used.

#### Non-phosphorylated neurofilaments (SMI32)

A mouse monoclonal antibody (IgG1), supplied as a high titer mouse ascites fluid, was used to detect intracellular neurofilaments, where it reacts with a non-phosphorylated epitope (clone 02-135; SMI32, Sternberger Monoclonals Inc., Lutherville, MD, USA; Sternberger and Sternberger, 1983). This antibody visualizes two bands (200 and 180 kDa) in conventional immunoblots (Goldstein et al., 1987).

#### Aggrecan (ACAN)

Perineuronal nets were detected with the monoclonal mouse anti-aggrecan antibody (Cat #: SM1353; RRID: AB_972582; Acris Antibodies GmbH, Herford, GERMANY), which was developed to identify human aggrecan protein, a proteoglycan component of the cartilage matrix (Matthews et al., 2002). A 1:75 dilution was used.

#### Chondroitin sulfate proteoglycan (CSPG)

PNs were detected with two antibodies directed against CSPG components: (1) a mouse monoclonal antibody (clone Cat-301; MAB5284, Chemicon) directed against a brain CSPG core protein, obtained with feline spinal cord fixed gray matter as immunogen and (2) a polyclonal rabbit antibody (Biogenesis, 2083–5,005 Poole, UK) raised against CSPG from bovine nasal cartilage and digested with chondroitinase ABC. It recognizes the antigenic determinants present on the sulfated glucuronic acid-N-acetyl-galactosamine disaccharide unmasked by chondroitinase ABC digestion (Härtig et al., 1994; Bertolotto et al., 1996). A 1:500 dilution was used in this study.

#### Voltage-gated potassium channel subunits Kv1.1 and Kv3.1b

The voltage-gated potassium channel subfamily A member 1 (KCNA1) subunit was detected with a polyclonal rabbit antibody (Cat #: APC-009; RRID: AB_2040144; Alomone Labs, Jerusalem, Israel). This antibody recognizes the intracellular Kv1.1 C-terminus epitope, corresponding to amino acid residues 416–495 of the mouse (*Mus musculus*) Kv1.1 protein. In this study, a 1:750 dilution was used.

The antibody against Kv3.1b amino acid residues 567–585, corresponding to the C-terminus of the voltage-gated potassium channel subunit KCNC1 (RRID: AB_2040166) was raised in rabbit (Weiser et al., 1995). In this study, a 1:6,000 dilution was used.

#### Low-voltage activated calcium channel subunits (Cav3.1, Cav3.2, Cav3.3)

Voltage-dependent T-type calcium channel subunits Cav3.1 (CACNA1G, α1G; Cat #: ACC-021; RRID: AB_2039779), Cav3.2 (CACNA1H, α1H; Cat #: ACC-025; RRID: AB_2039781) and Cav3.3 (CACNA1I, α1H; Cat #: ACC-009; RRID: AB_2039783) were detected with polyclonal rabbit antibodies from (Alomone Labs, Jerusalem, Israel). The Cav3.1 antibody recognizes intracellular amino acid residues 1–22 of the rat CACNA1G at the N-terminus. The Cav3.2 antibody recognizes amino acid residues 581–595 of the rat CACNA1H at the intracellular loop between domains D1 and D2. The Cav3.3 antibody recognizes amino acid residues 1,053–1,067 of the rat Cav3.3 between the intracellular domains II and III. In this study, a 1:1,000 dilution was used for all three antibodies.

#### Glutamate decarboxylase (GAD65/67)

GABAergic synaptic terminals were detected by a polyclonal rabbit anti-glutamate decarboxylase 65 & 67 (GAD65/67) antibody (Cat #: AB1511; RRID; AB_90715; Chemicon, Temecula, CA, USA), which recognizes C-terminus residues 572–585. GAD65 is membrane-anchored (585 a.a.) and is responsible for vesicular GABA production, whereas GAD67 is located in the cytoplasm (594 a.a.) and is responsible for a significant cytoplasmic GABA production. A 1:2,000 dilution was used.

#### GABA A receptor alpha 1

GABA receptor alpha subunit was detected by a polyclonal rabbit antibody (Cat #: 12410-1-AP; RRID; AB_2108692; Chromotek/Proteintech, Planegg/Martinsried, Bavaria, Germany), which recognizes an immunogen consisting of 260 aminoacids (21–280 aa encoded by BC030696). In this study, a 1:250 dilution was used.

#### Glycine receptor 1α

Glycine receptor 1α was detected by a monoclonal (clone mAb4a) mouse antibody (Cat #: 146011; RRID: AB_887722; Synaptic Systems, Göttingen, GERMANY), which recognizes amino acid residues 96–105 from the rat glycine receptor α1. A 1:300 dilution was used.

#### Vesicular glutamate transporters (vGlut1 and vGlut2)

The vesicular glutamate transporter 1 (vGlut1/SLC17A7) was detected with a polyclonal rabbit antibody (Cat #: 135303; RRID: AB_887875; Synaptic Systems, Göttingen, Germany). The vesicular glutamate transporter 2 (vGlut2/SLC17A6) was detected with a monoclonal mouse antibody (Cat #: MAB5504; RRID: AB_2187552; Chemicon, Temecula, CA, USA). Both, vGlut1 and vGlut2 mediate the uptake of glutamate into synaptic vesicles at the presynaptic nerve terminals of excitatory neurons, and usually show complementary expression patterns (Fremeau et al., 2004). In this study, a 1:3,000 dilution for vGlut1 and a 1:4,000 dilution for vGlut2 were used.

#### HCN channels

Hyperpolarization-activated cyclic nucleotide–gated channel subunit 1 (HCN1) was detected with polyclonal rabbit antibody (Cat #: PA5-78675; RRID: AB_2735891; Thermo Fischer Scientific, MA, USA), which recognizes a protein at the C-terminus region of human HCN1. A 1:400 dilution was used in this study.

HCN2 subunit was detected with polyclonal rabbit antibody (Cat #: PA5-77594; RRID: AB_2735892; Thermo Fischer Scientific, MA, USA), which recognizes the peptide (C)EEAGPAGEPRGSQAS, corresponding to amino acid residues 147–161 of human HCN2. In this study, a 1:1500 dilution was used.

HCN4 subunit was detected with polyclonal guinea pig antibody (Cat #: APC-052-GP (formerly AGP-004); RRID: AB_2340957; Alomone Labs, Jerusalem, Israel), which recognizes an intracellular epitope at the N-terminus, corresponding to amino acid residues 119–155 of human HCN4. In this study, a 1:200 dilution was used.

The specificities of all antibodies were validated first with primary antibody omission control and pre-absorption control tests (Mayadali et al., 2022).

## Results

### Delineation of omnipause neurons and cholinergic non-omnipause neurons in the nucleus raphe interpositus

#### Perineuronal nets as a reliable marker for identification of OPNs in human as well as in monkey

**Figure 1:**
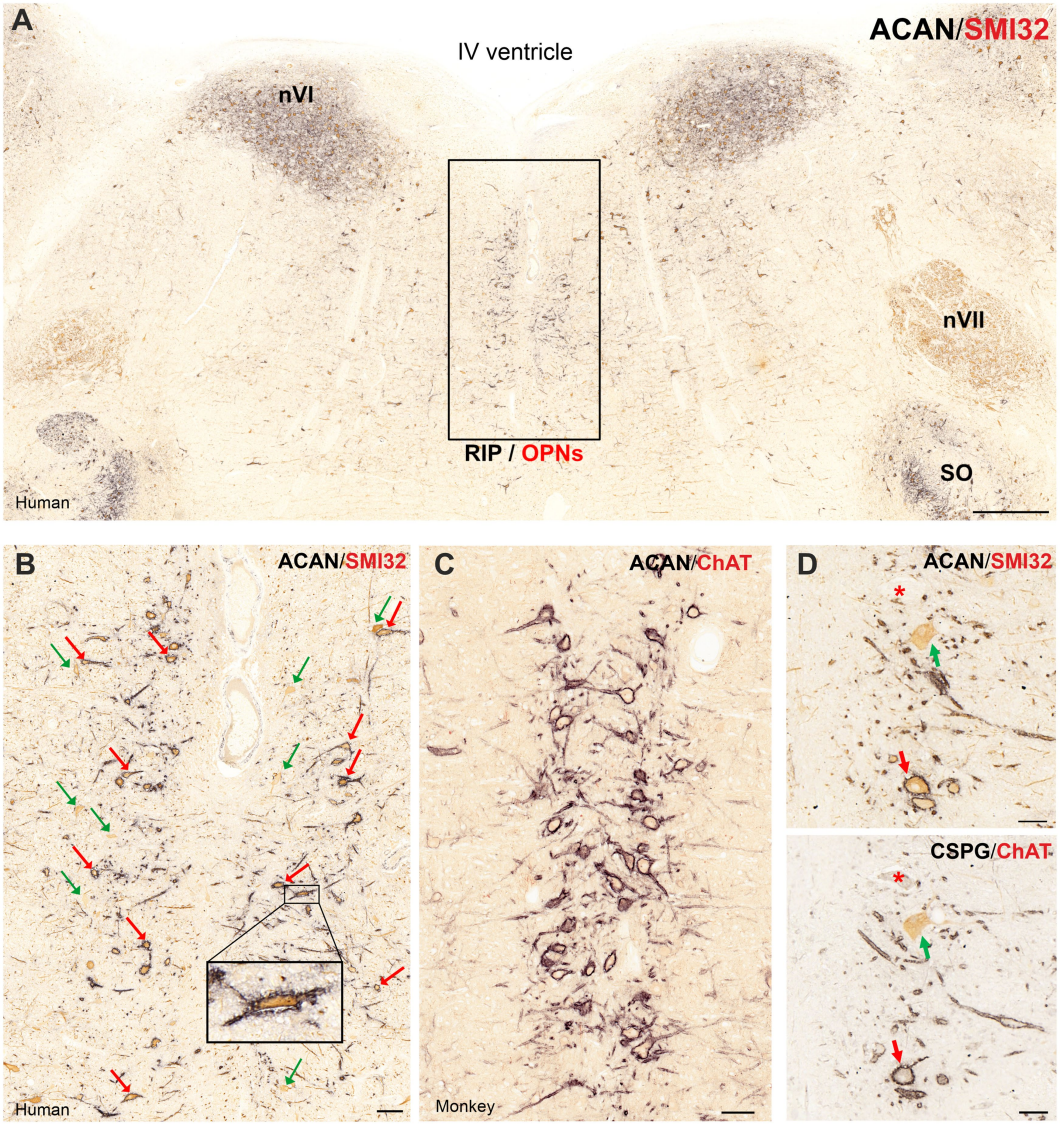
Immunoperoxidase-based histochemical classification of omnipause neurons (OPN) and cholinergic non-OPNs in human nucleus raphe interpositus (RIP). **(A)** Combined immunoperoxidase labeling of perineuronal net (PN) marker aggrecan (ACAN, black) and non-phosphorylated neurofilament marker SMI32 (brown) reveals OPNs that are arranged as two columns around the midline in human RIP. The box indicates the area illustrated at higher magnification in B. **(B)** Close-up demonstrating OPNs co-immunolabeled with ACAN and SMI32 (red arrows) Note the rather dispersed arrangement of OPNs around the midline compared to OPNs in monkey **(C)**. Also note several neurons that are not ensheathed by PN marker ACAN but express SMI32-immunolabelling (green arrows). **(C)** In monkey RIP, combined peroxidase labeling of PN marker ACAN (black) and ChAT (brown) demonstrates the absence of such cholinergic neurons and tightly organized OPN columns around the midline. **(D)** Combined peroxidase labeling of perineuronal net (PN) marker aggrecan (ACAN, black) and non-phosphorylated neurofilament marker SMI32 (brown) and PN marker CSPG (black) choline acetyltransferase (ChAT, brown) reveal the cholinergic non-OPN population (green arrow) that are not ensheathed by PNs. Scale bar represents 1 mm **A**, 100 μm **B,C** and 50 μm in **D**.

In human, double immunostaining of pontine sections using antibodies against the perineuronal net marker aggrecan (ACAN) or chondroitin-sulphate proteoglycan (CSPG) and non-phosphorylated neurofilament SMI32 highlighted a rather circumscribed neuron group around the midline at the level of the traversing rootlets of the abducens nerve, that are considered the saccadic omnipause neurons (Figure 1A) (Horn et al., 2003a; Horn and Adamcyzk, 2012; Eggers et al., 2015). The neurons displayed the characteristic morphology of saccadic omnipause neurons as medium-sized neurons with horizontally oriented long dendrites reaching across the midline (Figure 1B; red arrows, insert). However, close inspection of RIP in double-immunostained sections for SMI32 and ACAN or CSPG in human revealed a small consistent group of SMI32-positive neurons that lacked perineuronal nets. These SMI32-positive neurons showed similar size and morphology of putative OPNs and were scattered randomly throughout the extent of the OPN area (Figure 1B, green arrows). The double-immunostaining for choline acetyltransferase (ChAT) and ACAN of neighboring 5 μm thin sections showed that these SMI32-positive but ACAN-negative neurons are cholinergic and are therefore considered as a distinct cell group, different from glycinergic OPNs (Figure 1D, green arrows). They are hereafter referred to as cholinergic non-OPNs. Further, the inspection of sections from a previous project on the abducens nucleus (Mayadali et al., 2021) in monkey revealed that a consistent cholinergic population was not found in the OPN area of this species (Figure 1C), except few small neurons with different morphology occasionally found at different levels (not shown).

#### Quantitative analysis of OPNs and cholinergic non-OPNs

**Figure 2:**
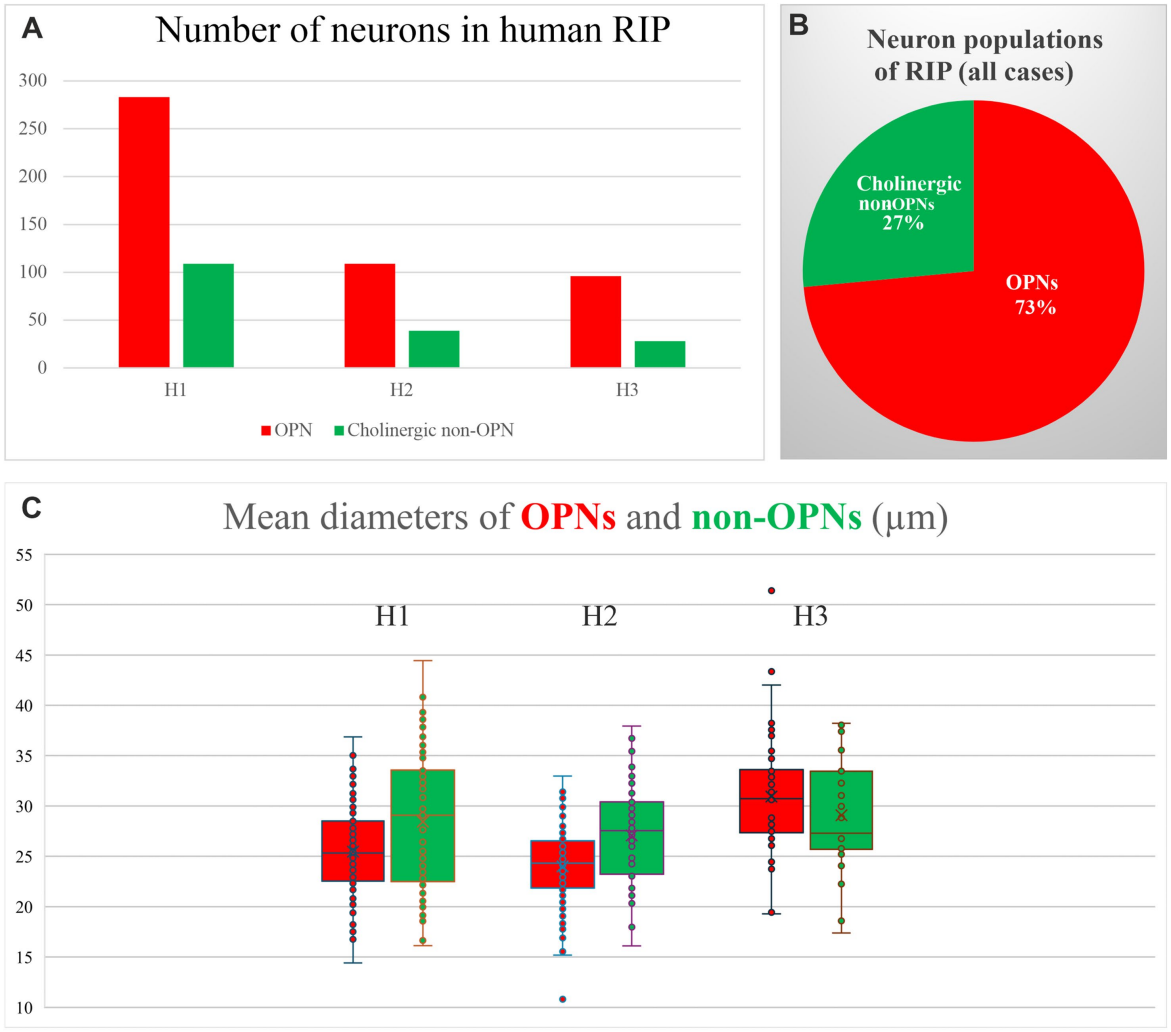
Quantitative analysis of OPN and non-OPN populations in human nucleus raphe interpositus in three cases (H1,H2, H3). **(A)** OPNs that are ensheathed by perineuronal nets (red bars) are more populous than the cholinergic non-OPNs (green bars). **(B)** Pie chart summarizing the proportion of OPNs and cholinergic non-OPNs within medium-sized neurons found in human RIP. **(C)** Box and Whiskers plot outlining the mean diameters of OPNs and cholinergic non-OPNs in human RIP. Morphological characteristics of OPNs and cholinergic non-OPNs are similar to each other. Note the somatic mean diameters for OPNs and non-OPNs do not differ significantly from each other, however, the variance in somatic size is somewhat larger in non-OPNs.

Further characterization of neurons in the RIP revealed that cholinergic non-OPNs consisted of roughly 1/4 to 1/3 of the medium-sized neurons with horizontally oriented dendrites within OPN area (Figure 2A). In Case 1 (female), only 22.6% of neurons were cholinergic non-OPNs, 27.8% in Case 2 (male) and 26.4% in Case 3 (female). The small sample size did not allow any conclusions to be drawn about sex differences. In total, 73.5% of all medium-sized neurons in RIP consisted of OPNs (Figure 2B).

Morphological analysis revealed that the cholinergic non-OPNs, on average, did not have significantly different diameters from those of the OPN population (Figure 2C). Two-tailed student’s *t*-test for two independent means yielded *p* values of *p* = 0.989 for H1, *p* = 0.3102 for H2 and *p* = 0.246 for H3; suggesting the mean diameters did not vary significantly between OPNs and cholinergic non-OPNs.

One common observation for all three cases was that the variance in mean diameter was higher in cholinergic non-OPNs compared to OPNs (Figure 2C). This was particularly apparent in the cases with larger datasets (H1&H2), as standard deviation of mean diameters for H1 showed 4.25 for OPNs, but 7.05 for non-OPNs; and for H2, standard deviation was 3.49 for OPNs, and 4.83 for non-OPNs.

**Figure 3:**
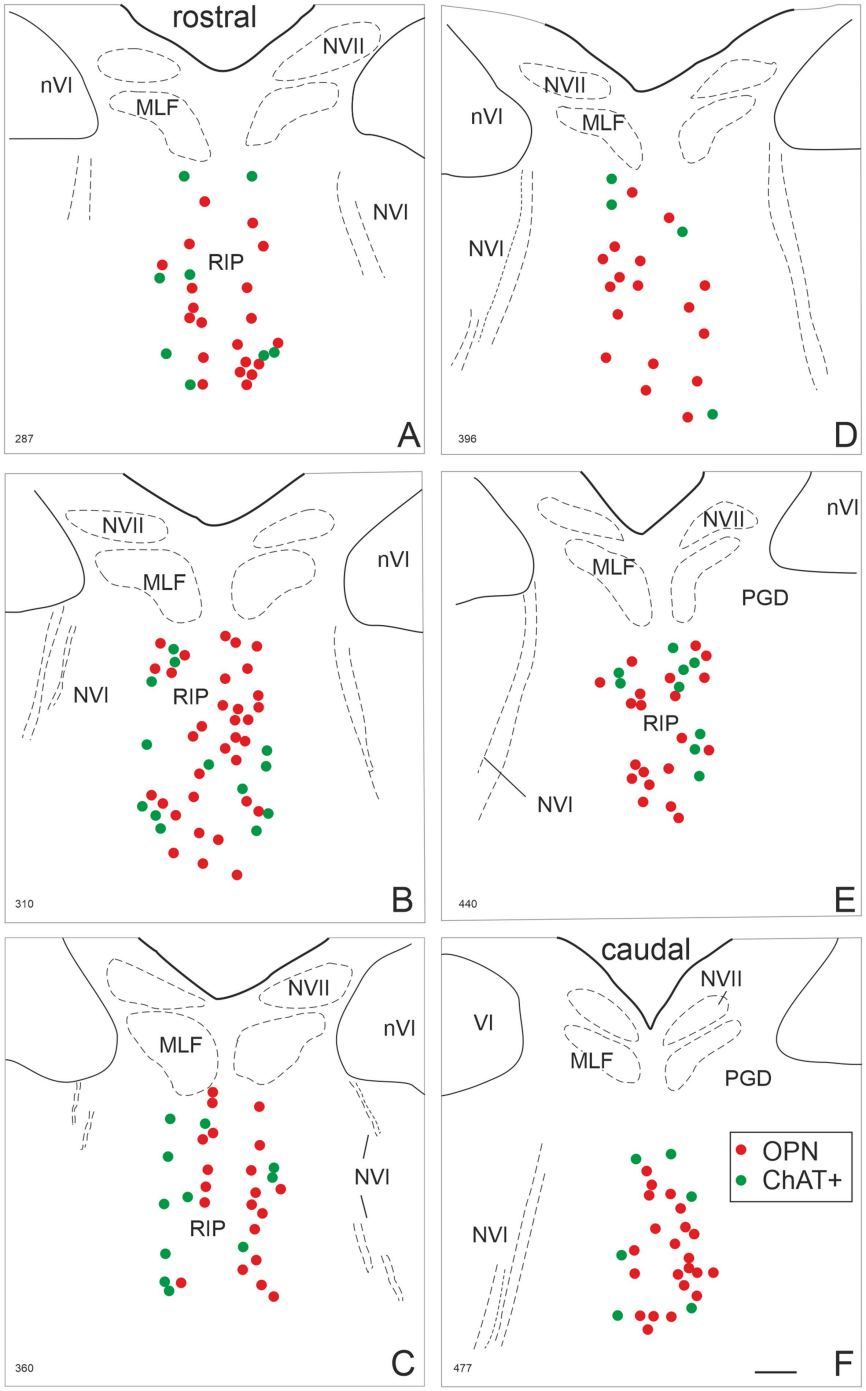
Localization of omnipause neurons (OPN) and cholinergic non-OPNs in human nucleus raphe interpositus (RIP) in one human case (H1). Plot of PN-positive OPNs (red dots) and PN-negative cholinergic non-OPNs within RIP at six pontine planes in the rostral **(A)** to the caudal **(F)** direction. This examination reveals that the cholinergic non-OPNs are not concentrated in either end of the rostro-caudal axis. However, cholinergic non-OPNs tended to lie more laterally to the midline than OPNs, as can be seen in C. MLF: medial longitudinal fasciculus; NVII: facial nerve; nVI: abducens nucleus; NVI: abducens nerve; PGD: nucleus paragigantocellularis dorsalis. Scale bar indicates 500 μm.

Next, the distribution of the cholinergic non-OPNs was evaluated along the rostro-caudal axis to determine whether non-OPNs tend to be concentrated at either end of the RIP area where OPNs are found. The plot of one exemplary human control case representing multiple planes of transverse pontine sections demonstrates that cholinergic non-OPNs can be found in comparable numbers at each level shown (Figure 3, green dots). In this specimen, perineuronal net bearing OPNs in RIP were found in a range of approximately 1.2 mm in rostro-caudal extension, and Figure 3 demonstrates transverse pontine layers covering approximately 1 mm within RIP. The cholinergic non-OPNs were found intermingled with OPNs (red dots) at varying distances from the midline with a slightly more frequent occurrence in the lateral parts of RIP (Figure 3).

### Distinct ion channel profiles in omnipause neurons and cholinergic non-omnipause neuron population

#### Voltage-gated potassium channels

**Figure 4:**
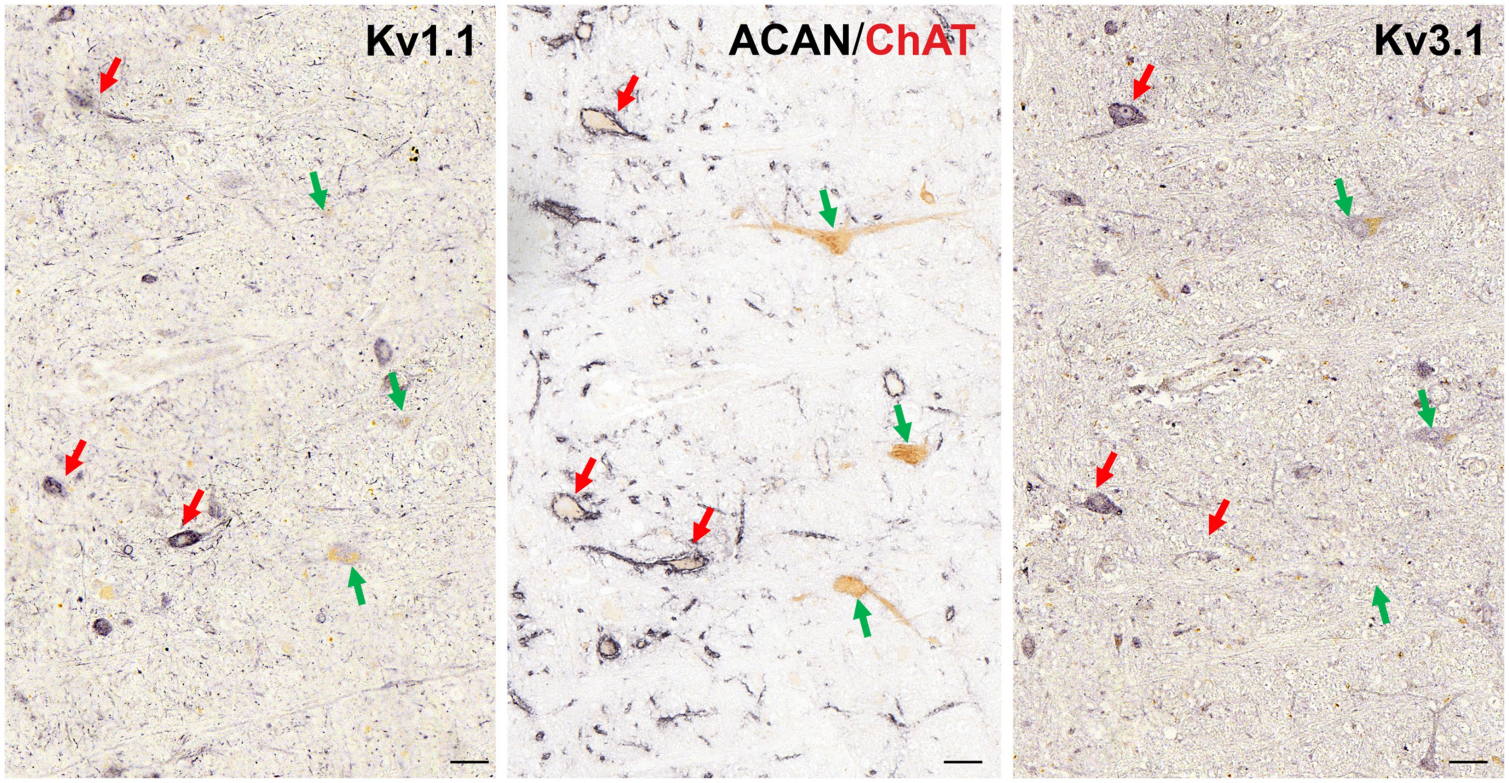
Immunoperoxidase labeling of Kv1.1 and Kv3.1b proteins in the neurons of human nucleus raphe interpositus (RIP). Consecutive 5 μm thick coronal paraffin sections through human RIP stained for Kv1.1 (left) and Kv3.1b (right). Note the stronger Kv1.1 immunolabeling in OPNs (red arrows) than in cholinergic non-OPNs (green arrows). Likewise, Kv3.1 subunit (right) is immunolabeled stronger in OPNs (red arrow) than cholinergic non-OPNs (green arrow). Scale bar represents 50 μm.

Previous studies established that OPNs that are characterized by combined immunolabeling for non-phosphorylated neurofilaments (SMI32) and perineuronal nets (PN) express the voltage-gated channel subunits Kv1.1 and Kv3.1. However, potential differences with the newly characterized cholinergic non-OPNs have not been examined. Therefore, 5 μm thick consecutive transverse paraffin sections of human RIP were immunostained with Kv1.1, ACAN/SMI32 and Kv3.1b antibodies, respectively, and were analyzed qualitatively (Figure 4). Immunostaining against Kv1.1 (Figure 4, left) and Kv3.1b (Figure 4, right) revealed that like the OPNs (red arrows), cholinergic non-OPNs (green arrows) are also equipped with these potassium channel subunits. However, both Kv1.1 (Figure 4 left) and Kv3.1b (Figure 4 right) immunolabeling in the OPNs was relatively stronger in comparison to cholinergic non-OPNs (Figure 4, right).

#### HCN channels

**Figure 5:**
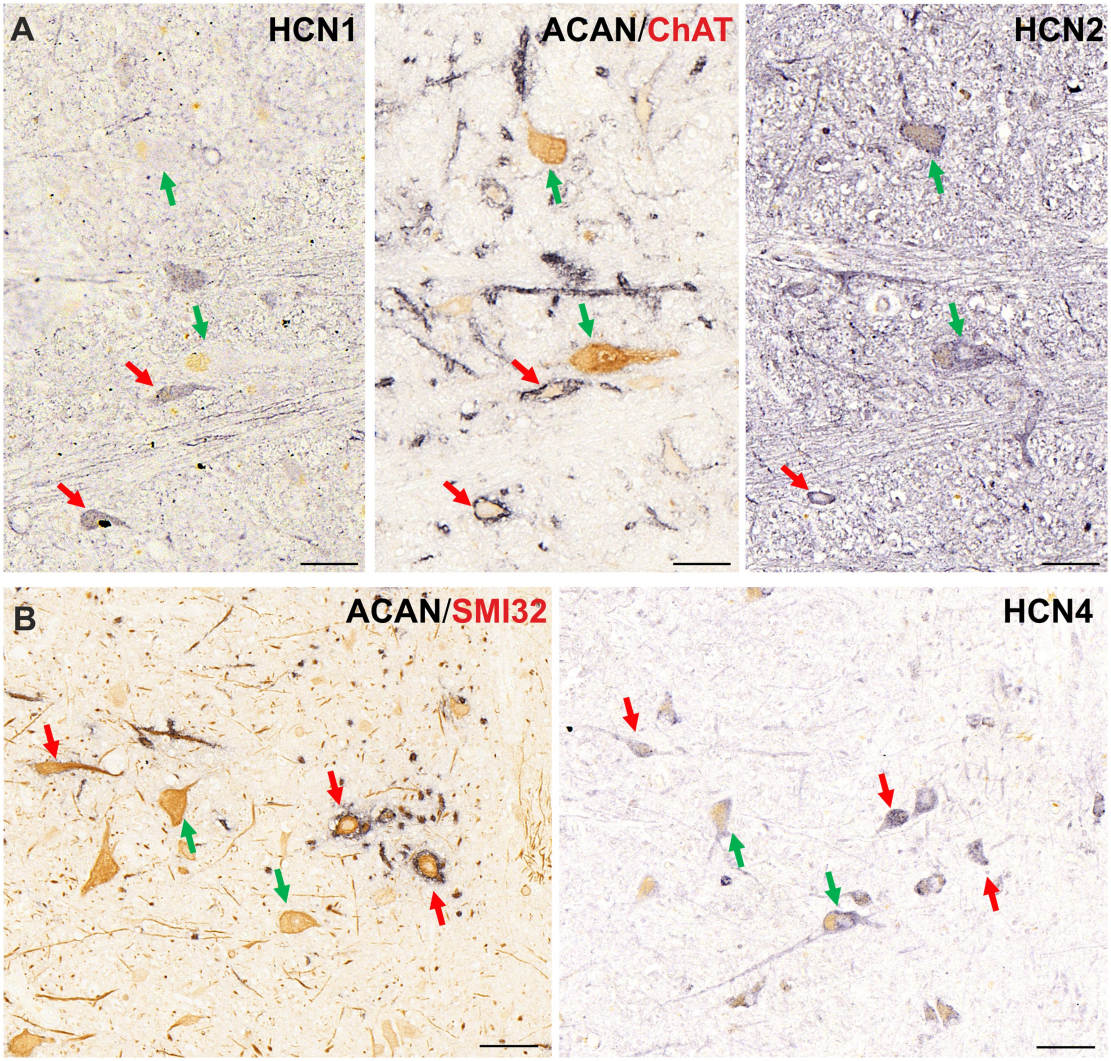
Immunoperoxidase labeling of HCN subunits in human nucleus raphe interpositus (RIP). **(A)** Consecutive 5 μm thick coronal paraffin sections through RIP stained for HCN1 (left) and HCN2 (right). HCN1 (left) is labeled only in OPNs, which are ensheathed by perineuronal nets (red arrows) and lacks completely in cholinergic non-OPNs (green arrows) (middle). On the other hand, HCN2 (right) immunolabeling is found in both neuron groups. **(B)** HCN4 immunolabeling (right) is ubiquitously found both in OPNs (red arrows) in cholinergic non-OPNs (green arrows). Scale bar represents 50 μm.

Other ion channel subunits that determine firing characteristics have not been previously studied in human OPNs. In order to determine the possible post-inhibitory firing characteristics of OPNs in human expression of hyperpolarization-activated cyclic nucleotide–gated (HCN) channels were investigated in human pontine sections and were compared to expression in non-OPNs (Figure 5). Close analysis of 5 μm thick consecutive transverse paraffin sections of human RIP revealed that the HCN1 subunit was expressed exclusively in the OPNs defined by PN ensheathment (Figure 5A left, red arrows), but spared the cholinergic non-OPNs (Figure 5A left, green arrows). On the other hand, equally strong HCN2 immunolabeling was observed within the OPNs with PN ensheathment and in cholinergic non-OPN population (Figure 5A right, red and green arrows, respectively). Finally, HCN4 subunit was found ubiquitously in both OPNs, and cholinergic non-OPNs (Figure 5B, right).

#### T-type Ca^2+^ channels (Cav3)

**Figure 6:**
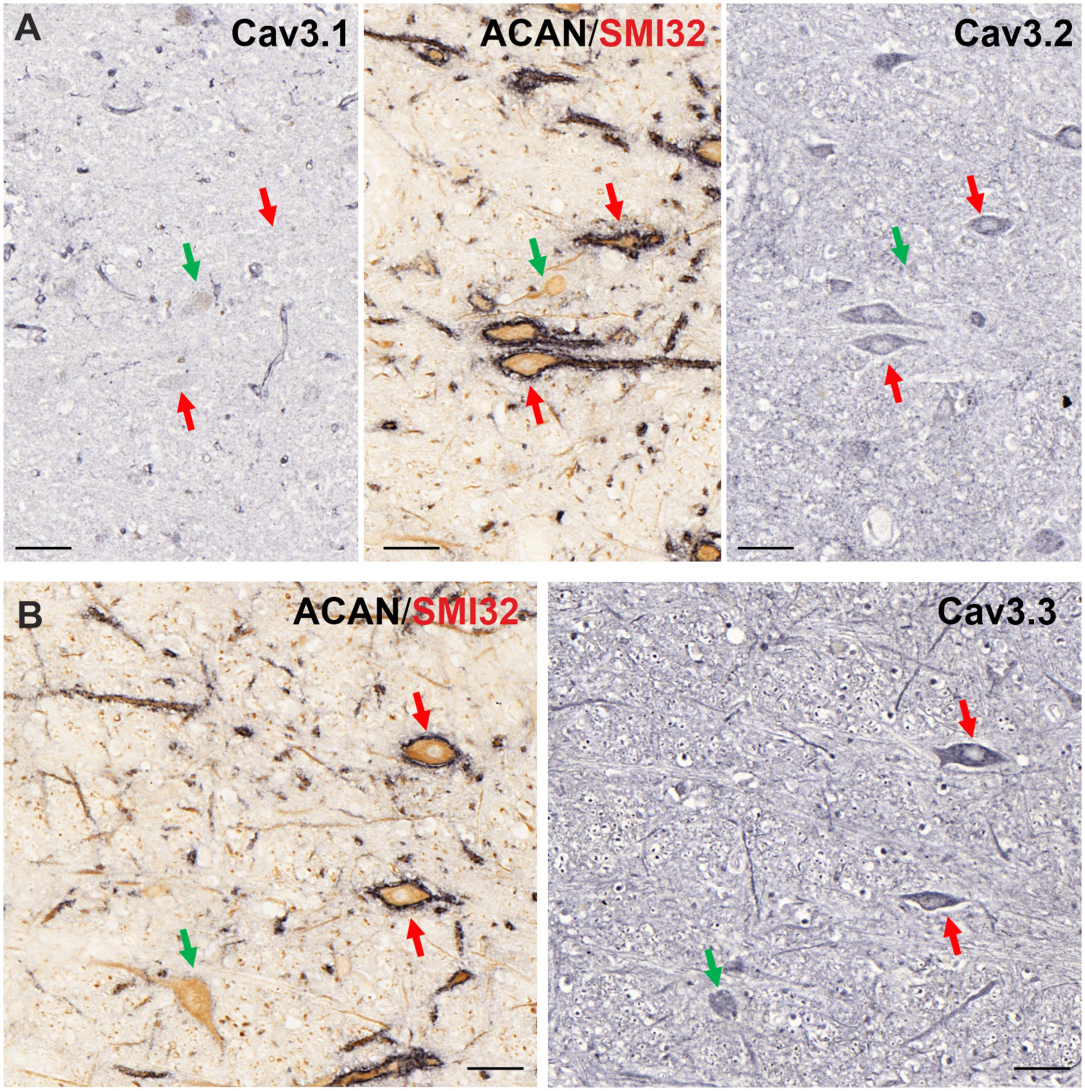
Immunoperoxidase labeling of Cav3 subunits in neurons of human nucleus raphe interpositus (RIP). **(A)** Consecutive 5 μm thick coronal paraffin sections through RIP stained for Cav3.1 (left) and Cav3.2 (right). Cav3.1 (left) is only weakly expressed in both neuron groups with variation. On the other hand, Cav3.2 (right) immunolabeling is found only in OPNs, which are ensheathed by perineuronal nets (red arrows) and is absent in cholinergic non-OPNs (green arrow) (middle). **(B)** Cav3.3 immunolabeling (right) is ubiquitously found in both OPNs (red arrows) and cholinergic non-OPNs (green arrows). Scale bar represents 50 μm.

Another low voltage-activated ion channel family that determines post inhibitory rebound behavior are Cav3 channels, which were also studied in OPNs and cholinergic non-OPNs in human (Figure 6). Whereas Cav3.1 subunit was not found in either of the populations (Figure 6A), Cav3.2 and Cav3.3 immunolabeling was each detected in both OPNs, and cholinergic non-OPNs (Figure 6A, right; B, red and green arrows, respectively).

### Mutually exclusive transmitter-related protein expression in omnipause neurons and cholinergic non-omnipause neurons

#### Inhibitory (GABA/glycinergic) transmitters

**Figure 7:**
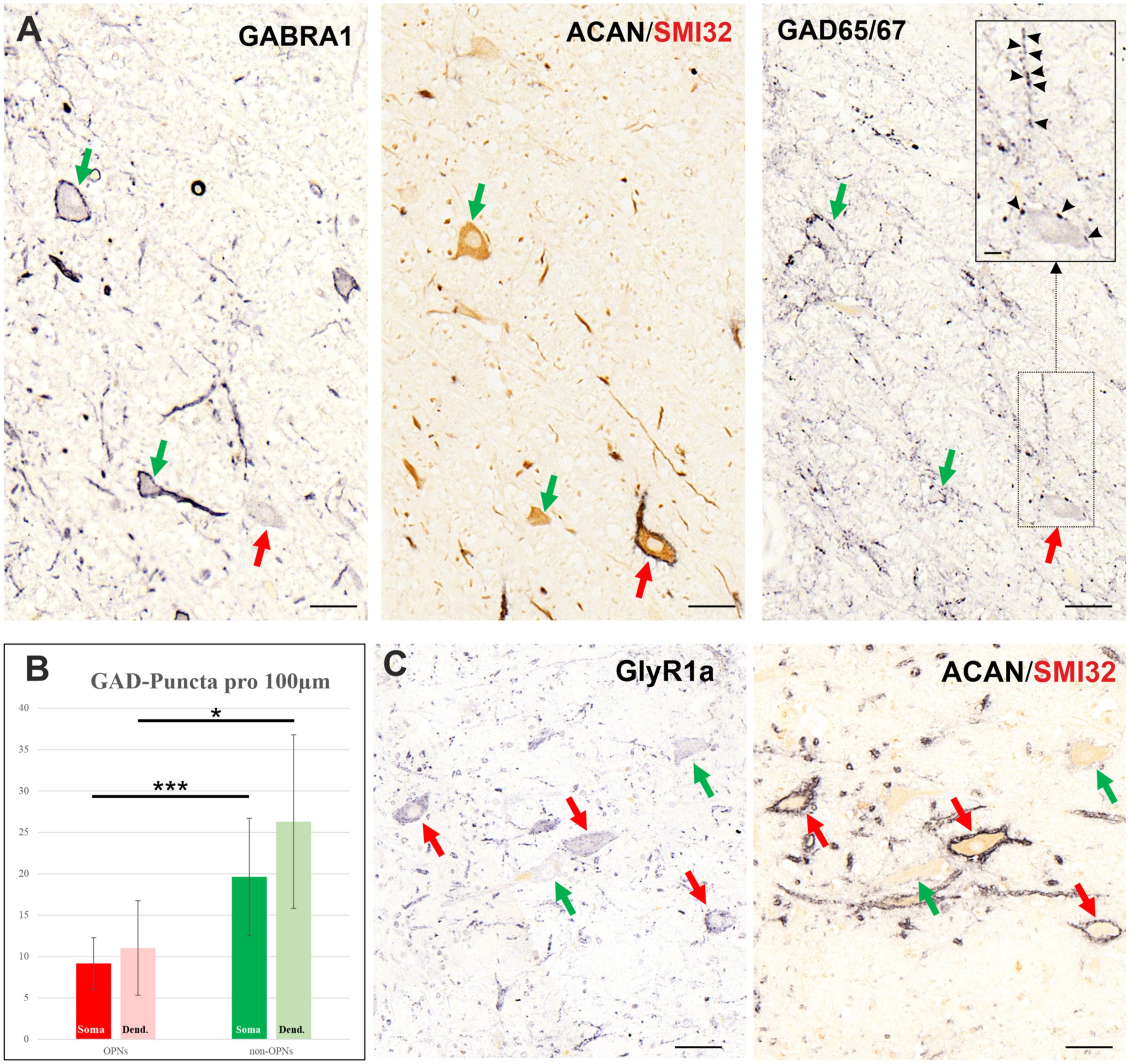
Inhibitory transmitters in neurons of human nucleus raphe interpositus (RIP). **(A)** Consecutive 5 μm thick coronal paraffin sections through RIP stained with GABA A receptor (GABRA1, left) and glutamate decarboxylase (GAD65/67, right) antibodies. Note that cholinergic non-OPNs (green arrows) express intense GABRA1 immunoreactivity compared to OPNs’ (red arrows) weak labeling on their somatic membrane. This trend is reflected in the dense GABAergic inputs shown by GAD65/67 immunolabeling (right) to the cholinergic non-OPNs (green arrows) and sparse inputs to somatic membranes of OPNs (red arrows). The density difference between somatic and dendritic labelling of OPNs can be seen in the inset on the top-right corner of the image (black arrowheads). The close up (inset) is magnified from the boxed region. **(B)** Quantitative analysis of the GAD65/67 immunopositive puncta on somatic (dark-colored bars) and dendritic (light-colored bars) membranes of OPNs (red) and cholinergic non-OPNs (green) demonstrating significantly denser GABAergic inputs to non-OPNs. **(C)** Glycine receptor 1a immunolabeling in neurons of RIP demonstrate a complimentary pattern to GABRA1. Note that cholinergic non-OPNs (green arrows) express weak GlyR1 immunoreactivity compared to OPNs’ (red arrows) intense labeling on their somatic membrane. Scale bars represent 50 μm, in A and C; 10 μm in the inset in right column (GAD) of A. ***p < 0.05, *p < 0.1.

The examination of GABA related protein expression in human pontine sections revealed distinct profiles regarding GABAergic inputs and the receptors in the OPNs and cholinergic non-OPNs (Figure 7). The immunostaining against glutamate decarboxylase (GAD65/67) and GABA A receptor 1 (GABRA1) antibodies performed in consecutive 5 μm sections revealed that the somata of OPNs are associated with less GABA-related proteins compared to cholinergic non-OPNs (Figures 7A,B). Specifically, the quantitative investigation of the GABAergic inputs to these neurons visualized by presynaptic GAD-positive puncta yielded 9.17 puncta per 100 μm of the somatic membranes in the OPNs (*n* = 21), and ~19.62 puncta per 100 μm in the cholinergic non-OPNs (*n* = 15) (Figure 7B). This difference was statistically significant as demonstrated by student’s *t*-test at *p* < 0.05 (*p* = 0.008). Although not thoroughly evaluated, as the low section thickness often prevented a clear assignment of dendrites to OPNs, a higher density of GAD inputs was observed in analyzable dendrites (Figure 7A right, red arrow). Due to low number of available samples, only a low number of measurements for the dendritic GAD input was possible (*n* = 9 for both OPNs and non-OPNs), the difference was significant only at *p* < 0.1 (*p* = 0.086). The differences in GAD input density between the somatic and dendritic membranes of OPNs or cholinergic non-OPNs also did not yield statistically significant values (*p* > 0.1).

The difference between somatic GABAergic input density was also reflected in the postsynaptic membrane labelling of GABA A receptor 1 (Figure 7A left). While OPNs expressed low GABRA1 labelling on their somatic membrane, cholinergic non-OPNs had strong immunolabeling on their somatic membrane (Figure 7A left; red and green arrows, respectively). In line with the observation of presynaptic GAD-positive puncta, GABRA1 labelling strength in OPNs was higher on the membranes of their dendrites (Figure 7a left, red arrow, right, black arrowheads).

Interestingly, glycine receptor 1a was expressed in a complementary manner to GABA receptor (Figure 7C). While GlyR1 labelling was prominent in OPNs (red arrows), which are themselves glycinergic, cholinergic non-OPNs lacked strong expression of GlyR1 protein on their membrane (Figure 7C, green arrows).

#### Excitatory (glutamatergic) inputs and receptors

**Figure 8:**
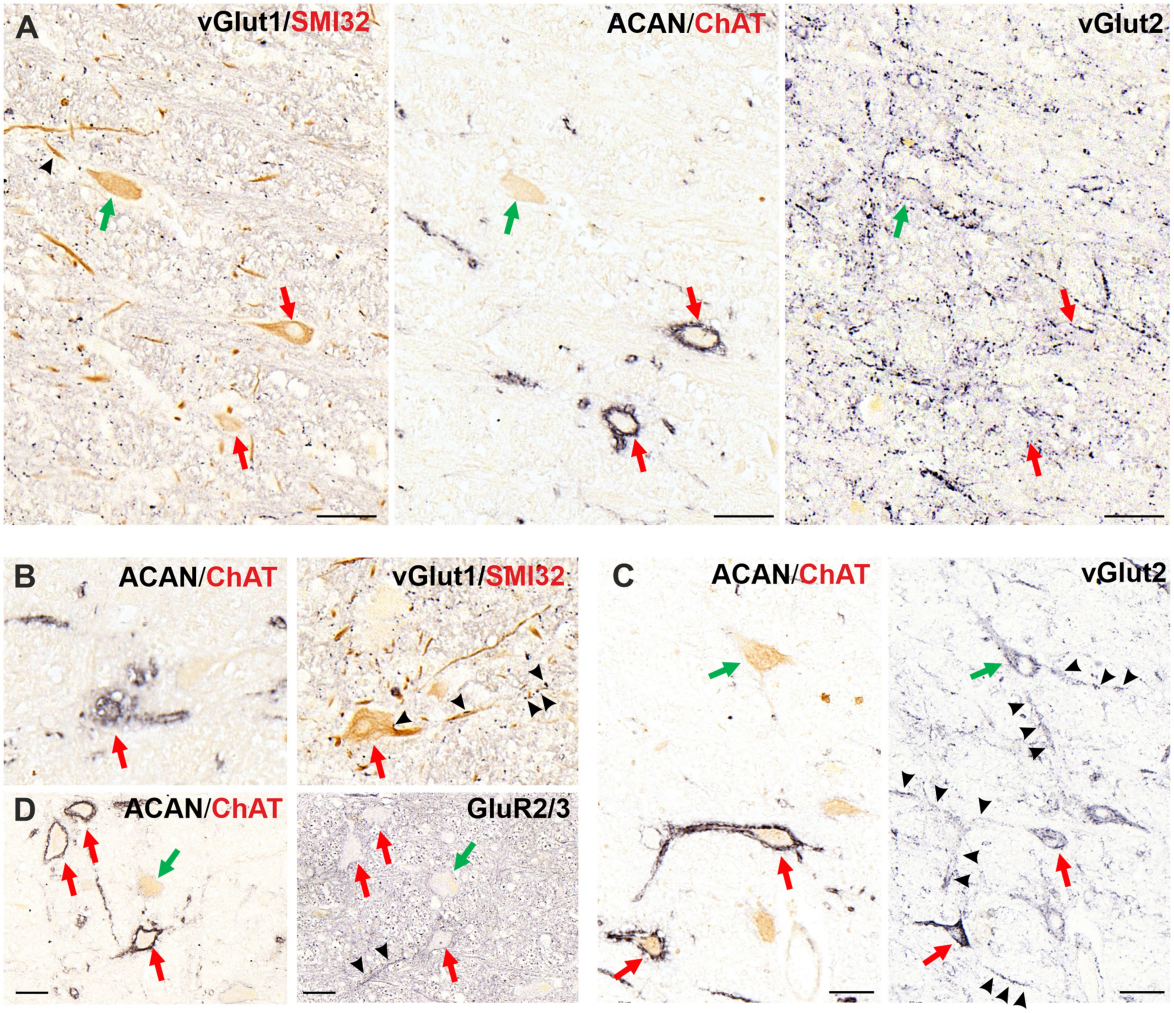
Glutamatergic excitatory inputs and receptors in the omnipause neurons (OPNs) and cholinergic non-OPNs in human nucleus raphe interpositus (RIP). **(A)** Consecutive 5 μm thick coronal paraffin sections through RIP stained with vesicular glutamate transporters 1 (left) and 2 (right) antibodies. Both OPNs (red arrows) and cholinergic non-OPNs (middle, green arrow), are contacted rarely by vGlut1-positive puncta on their somata (left, black arrowhead). However, vGlut2-positive puncta are more abundant in RIP (right), and are also found on somata of both OPNs and non-OPNs. **(B)** vGlut1- positive labelling is encountered at the dendrites of OPNs (red arrow, black arrowheads) and cholinergic non-OPNs (A left; green arrow, arrowheads). These puncta (arrowheads) are found at proximal dendrites and at distal dendrites. **(C)** A similar labelling pattern is observed with vGlut2-positive puncta for both OPNs (red arrow) and cholinergic non-OPNs (green arrow), and can be seen indicated with black arrowheads. **(D)** Immunostaining with the glutamate receptor 2/3 antibody yields similar labelling on somatic membranes of both OPNs (red arrows) and non-OPNs (green arrows). The labelling is more intense in the dendrites of OPNs (black arrowheads) for the glutamate receptors, similar to glutamatergic inputs. Scale bars represent 50 μm.

Lastly, as the main excitatory transmitter, glutamate inputs and ionotropic AMPA receptor subunits GluR2/3 were examined to the OPNs and cholinergic non-OPNs (Figure 8). Immunohistochemistry of vGlut1 in 5 μm thick consecutive transverse paraffin sections of human RIP revealed that OPNs, as well as cholinergic non-OPNs receive virtually no vGlut1 immunopositive puncta on their somatic membranes (Figure 8A, left), but both were contacted by vGlut1 immunopositive puncta on their dendritic membranes (Figures 8A,B,C black arrowheads). The labelling density appeared to increase with the distance from soma. On the other hand, vGlut2 was encountered more abundantly within the RIP (Figure 8A, right; 8C right). vGlut2-immunopositive puncta were observed in contact with the somatic membrane of both OPNs (red arrows) and cholinergic non-OPNs (green arrow). Cholinergic non-OPNs were contacted by higher density of vGlu2-immunopositive puncta (Figure 8A, C, right, green arrow, black arrowheads). Similar to vGlut1 immunolabeling, the density of vGlut2-positive puncta seemed to increase with the distance to the somatic membrane (Figure 8C, black arrowheads) for both OPNs (red arrows) and non-OPNs (green arrow).

The glutamate receptor GluR2/3 immunolabeling demonstrated a similar pattern with the vGlut2-positive puncta density (Figure 8D). The somatic membranes of both OPNs (red arrows) and cholinergic non-OPNs (green arrow) were immunolabeled by GluR2/3 antibody, however, the density increased in the dendritic membranes (Figure 8D right, black arrowheads).

## Discussion

This study investigated ion channel and transmitter-related profiles of saccadic omnipause neurons (OPN) in human nucleus raphe interpositus (RIP), which were delineated from a novel, cholinergic non-OPN population not present in monkey. Both neuron populations expressed immunoreactivity for SMI32, the voltage-gated potassium channel subunits Kv1.1 and Kv3.1b, but only OPNs were enwrapped by prominent perineuronal nets, which were absent from non-OPNs. OPNs expressed low-voltage activated ion channels Cav3.2 and Ca3.3 and HCN1, HCN2 and HCN4 supporting post-inhibitory rebound, whereas non-OPNs lacked HCN1-channels. Further, both neuron populations in RIP displayed significant differences in the presence and density of transmitter inputs. Whereas a rather similar supply of glutamatergic terminals dominated by vGlut2 input is present at both groups, a completely reversed pattern was found for inhibitory transmitters. Postsynaptic glycine receptors were only found in OPNs, whereas non-OPNs received a strong GABA input in contrast to a weak input to OPNs. Again, the complementary patterns of inhibitory inputs to both cell groups within RIP support the assumption that the non-OPNs represent a distinct population different from saccadic OPNs.

Thus, the present study demonstrates the presence of required ion channels in human OPNs that enable the high firing rates that are rapidly recovered after inhibition for the switch between fixation and saccades. Further, this is the first description of transmitter-related proteins associated with OPNs in human supporting existing animal data on the saccade generation circuit. The data will provide the basis for the investigation of post-mortem clinical cases with saccadic disorders.

### Identification of cholinergic non-OPN population in human RIP, not present in monkey

The histological and morphological organization of OPNs in human differ from monkey. In monkey nucleus raphe interpositus (RIP), the neurons are found as two relatively neat vertical cell columns on either side of the midline (Büttner-Ennever et al., 1988; Horn et al., 1994), which evidently all represent OPNs. This is suggested by electrophysiological recordings showing that except few, all identified OPNs were found in RIP clustered together assuming that RIP consists solely of OPNs (Langer and Kaneko, 1990). In accordance with these observations, all neurons in RIP of monkey show a characteristic histochemical staining pattern for non-phosphorylated neurofilament marker SMI32, calcium-binding protein parvalbumin (PAV), cytochrome oxidase and strong perineuronal net (PN) labelling (Büttner-Ennever et al., 1988; Horn et al., 2003a; Horn, 2006). Together with anatomical location and characteristic neuron morphology, the histochemical pattern was used to identify the OPNs in other mammals as well, including human (Büttner-Ennever et al., 1988; Horn et al., 1994; Horn et al., 2003a; Hittinger and Horn, 2012). However, the same immunostainings for PNs, PAV and SMI32 reveal that putative OPNs with similar morphology in human are not located in a neatly organized column structure equidistant from the midline, but more scattered around the midline (Horn et al., 2003a; Horn and Adamczyk, 2012). Moreover, in human the immunostaining for PN marker aggrecan (ACAN) or chondroitin-sulfate proteoglycan (CSPG) combined with SMI32 and choline acetyltransferase (ChAT) revealed a cholinergic neuronal population within RIP that had similar morphological characteristics as OPNs (i.e mid-sized neurons with dendrites extending bilaterally in a horizontal axis). Although intermingled with OPNs, several properties suggest that these cholinergic neurons can be classified into a distinct population different from OPNs: OPNs are inhibitory and use glycine as a transmitter as shown by different independent methods (Nakao et al., 1988; Horn et al., 1994), whereas the ChAT-positive neurons do not express glycine-related proteins. Like OPNs, the ChAT-positive neurons were SMI32-positive in support for projection neurons, but unlike OPNs, they lack PNs suggesting different firing characteristics.

Interestingly, a similar group of ChAT-positive neurons as seen in human RIP is not present in monkey brainstem (own observations; Mesulam et al., 1984). Therefore animal recording and tract-tracing studies cannot contribute any information about connections and function of Chat-positive non-OPNs within human RIP, which only allows for speculative propositions at this point.

There are only few systematic studies on cholinergic neurons in the human reticular formation: Mesulam and coauthors have described six cholinergic groups in rat, monkey and human, and numbered them from rostral to caudal Ch1-Ch6 (Mesulam et al., 1989). Only groups Ch5 and Ch6 lie as a continuum in the prepontine tegmentum and include the pedunculopontine nucleus (Ch5) and the laterodorsal tegmental nucleus (Ch6). ChAT-positive neurons of groups Ch1-Ch4 subnuclei in the magnocellular basal forebrain lack PNs, unlike the adjacent PAV- and GABA-positive neurons with high firing rates (Adams et al., 2001). There, it is assumed that PN-lacking ChAT-positive neurons may represent projection neurons with slow modulatory functions (Mesulam, 1995; Zaborszky et al., 1999). Accordingly, the ChAT-positive neurons within the human RIP may just be part of the ChAT-positive neuron population in the prepontine tegmentum that are scattered throughout the medullary and pontine reticular formation. They may represent projection neurons with far reaching axons as indicated by their expression of non-phosphorylated neurofilaments (SMI32-staining) (Fuentes-Santamaria et al., 2006) exerting modulatory actions on their target neurons. Acetylcholine has been shown to be a major neuromodulator with central role in attention, movement and behavioural flexibility as described for ChAT neurons of the pedunculopontine nucleus and laterodorsal tegmental nucleus, which provide widespread innervation to the thalamus and basal ganglia (Mena-Segovia et al., 2008; Dautan et al., 2016). Further, cholinergic neurons of Ch5 and Ch6 are involved in the initiation of REM-sleep characterized by rapid eye movements using the same premotor network for saccades in awake state (Brown et al., 2012; Fraigne et al., 2015; van Dort et al., 2014). Injections of acetylcholine agonists into the pontomedullary brainstem induce REM sleep possibly mimicking the action of Ch5 and Ch6 cell groups (Márquez-Ruiz and Escudero, 2010). Since REM sleep is present in most animals (Peever and Fuller, 2017) with only minor differences of REM sleep pattern between macaque monkey and human (Hsieh et al., 2008), the cholinergic non-OPNs found only in human are unlikely to be involved in REM sleep circuits.

### Biophysiological and functional implications of distinct ion channel between OPNs and cholinergic non-OPNs

As thoroughly discussed in our previous works (Horn et al., 2018; Mayadali et al., 2021; Mayadali et al., 2022; Mayadali et al., 2024), the density of perineuronal net (PN) ensheathment influences neuron firing characteristics both directly and indirectly (Kudo et al., 2023). This occurs by (co)regulating the content of neurotransmitters and ion channels in neuronal membranes, as well as by affecting the expression of calcium-binding proteins in neurons (Yamada et al., 2015; Wingert and Sorg, 2021). For example, it is known that PN density can impact biophysical properties like high firing rates by co-regulating ion channel subunits associated with high firing capacity, such as Kv1.1 and Kv3.1 (Härtig et al., 1999; Favuzzi et al., 2017). Therefore, PN-ensheathment (alongside parvalbumin expression) could be used to assess the high metabolic needs of a neuron population (Favuzzi et al., 2017). We observed that the lack of PNs in cholinergic non-OPNs was accompanied by a weaker Kv3.1b immunolabeling (Figure 1). This implies that cholinergic non-OPNs could lack the machinery to sustain high firing rates compared to OPNs, which can fire up to 200 Hz (Henn et al., 1984). The usage of acetylcholine as a transmitter alone cannot be the cause for the absence of PNs, since they are known to enwrap cholinergic motoneurons of the oculomotor (Jäger et al., 2013; Horn et al., 2018; Sánchez-Ventura et al., 2022; Mayadali et al., 2024) or skeletal system (Jäger et al., 2013; Sánchez-Ventura et al., 2022).

The high frequency discharge of neurons, such as OPNs, not only depends on the fast-firing capacity enabled by Kv channels, but also requires the presence of low-voltage activated cation channels that govern effective recovery from hyperpolarized voltages, known as post-inhibitory rebound (PIR) phenomenon (Enderle and Engelken, 1995). Here we showed for the first time that the OPNs in human contain low voltage-activated cation channels of the HCN and Cav3 channel families as the required machinery for PIR, which is defined as membrane depolarization evoked at the offset of a prior hyperpolarizing stimulus resulting in an enhanced or patterned spike output (Dodla, 2013). OPNs differed in low voltage-activated cation channel expression profile compared to cholinergic non-OPNs, as HCN1-immunolabelling was only present in OPNs, which indicates differences in neuronal excitability and readiness for post-inhibitory rebound (PIR) bursting of both populations. Shortly before and during a saccade the tonic activity of OPNs is inhibited, and resumed rapidly, when the eyes fixate on a new target (Cohen and Henn, 1972; Luschei and Fuchs, 1972; Keller, 1974; Evinger et al., 1982).

### Transmitter-related proteins in human RIP

According to long-standing hypotheses the inhibitory OPNs provide the primary gating mechanism for the generation of saccades. This requires an excitatory input that drives OPN tonic activity during fixation and an inhibitory input that releases premotor burst neurons from OPN inhibition for saccade initiation (Robinson, 1975). In the following sections we will discuss the profiles of transmitter-related proteins in OPNs in the context of the known pathways for saccade generation circuits and compare it to non-OPNs in RIP.

#### Excitatory inputs to RIP

Electrophysiological recording studies in cat and monkey revealed that the intermediate layers of the rostral pole of the superior colliculus representing the fovea contains neurons with tonic activity during fixation (Munoz and Wurtz, 1993a, 1993b; Everling et al., 1998). These neurons send monosynaptic excitatory projections by way of the crossing fibers of the predorsal bundle to the OPNs (Pare and Guitton, 1994; Takahashi et al., 2022). Anatomically, this projection was shown in monkey by the presence of rather large tracer-labeled terminals around OPN cell bodies following ^3^H-leucine injections into the rostral pole of the superior colliculus (Büttner-Ennever et al., 1999). As previously shown for monkey with glutamate antibodies (Horn et al., 1994), the human OPNs receive a glutamatergic input here demonstrated by immunostaining for the vesicular glutamate transporters vGlut1 and vGlut2. These transporters are involved in the transfer of glutamate from the cytoplasm into synaptic vesicles but appear to be linked to distinct glutamatergic synapses differing in the transmitter release probability (Fremeau et al., 2001; Fremeau et al., 2004). In line with this, in the visual system vGlut2 is found in synapses which significantly “drive” neural activity in their postsynaptic targets, whereas vGlut1 is found in synapses governing modulatory inputs with small EPSPs and show low transmitter release probability (Sherman and Guillery, 1998; Fremeau et al., 2004; Balaram et al., 2013). The deep layers of the superior colliculus in cat and monkey (prosimian galago) contain large neurons with strong glutamate or vGlut2 mRNA expression, respectively, similar to parvalbumin-positive neurons projecting in the predorsal bundle (cat) and may correspond to large X-cells with thick axons (Moschovakis and Karabelas, 1985; Balaram et al., 2013). Accordingly, the (rather large) vGlut2-positive boutons found at human OPNs in this study may contribute to the direct projection from large X-cells from the fixation zone of the superior colliculus, which excite OPNs during fixation (Pare and Guitton, 1994; Munoz and Wurtz, 1995). As a driving input vGlut2-positive terminals may also include the more distributed terminals at OPNs originating from T-cells with thinner axons in the “small saccade zone” of the superior colliculus possibly mediating microsaccades (Moschovakis and Karabelas, 1985; Scudder et al., 1996; Büttner-Ennever et al., 1999; Hafed et al., 2009). This is in line with the presence of vGlut2 mRNA expression in neurons of the deep layers of the superior colliculus, which lack vGlut1mRNA positive neurons (Balaram et al., 2013). The primarily dendritic vGlut1input to OPNs could derive from cortical areas, such as frontal eye fields, since neurons expressing vGlut1 mRNA are mainly found in the cortex (Segraves, 1992; Balaram et al., 2013).

Previous work in monkey revealed that the dendrites of OPNs receive a considerable amount of orexin A positive terminals (Schreyer et al., 2009). The neuropeptide orexin A is expressed by a discrete population of neurons within the lateral, dorsomedial and perifornical hypothalamus (Williams et al., 2022) and exerts excitatory functions via widespread projections involving multiple physiological functions including the promotion of wakefulness and suppression of REM sleep (Thannickal et al., 2000; Sakurai, 2007). Double-labelling studies in the rat revealed that at least 50% of orexin-positive neurons in the hypothalamus expressed vGlut2 mRNA, and a small fraction vGlut1 (Rosin et al., 2003). Therefore it is very likely that vGlut1 and/or vGlut2-positive terminals at OPN dendrites include orexin-positive inputs that provide an excitatory drive or modulation to OPNs in wakefulness (Schreyer et al., 2009). This is supported by recording experiments in monkey, where OPN activity ceased during sleep but resumed their firing activity at the sleep–wake transition (Henn et al., 1984).

Only recently a distinct calbindin-positive cell group termed nucleus papilio was identified within the dorsal paragigantocellular nucleus of different mammalian species, which are active during REM sleep (Gutierrez Herrera et al., 2019). Optogenetic activation of these neurons in transgenic mouse model induced eye movements selectively during REM sleep, which were suppressed after genetic ablation. Furthermore, the calbindin-positive neurons express vGlut2 and project to motoneurones and premotor neurons of the saccadic circuitry including RIP (Gutierrez Herrera et al., 2019), which still has to be proved in primates. Thus, the medullary calbindin-positive neurons may well contribute to vGlut2-positive input to OPNs seen in the present study to interact with the saccade generator for rapid eye movements during sleep (Vanni-Mercier et al., 1994; Márquez-Ruiz and Escudero, 2010).

Since cholinergic non-OPNs show a similar pattern and distribution of vGlut1 and vGlut2 inputs, they may receive glutamatergic afferents from similar sources as OPNs.

#### Inhibitory inputs to RIP

During fixation the OPNs exert a tonic inhibition by glycine on premotor burst neurons (Horn et al., 1994; Iwamoto et al., 2009; Kanda et al., 2007), which must be released by an inhibitory trigger input to OPNs to start a saccade in any direction. Recording studies showed that 10–12 msec prior to a saccade OPNs cease firing, remain off for the duration of the saccade, and resume tonic firing at the end of the saccade promoting fixation (Robinson, 1975; Iwamoto et al., 2009; Rucker et al., 2011). The resumption of OPN discharge determines the end of a saccade as indicated by the abrupt stop of saccades in mid-flight after electrical stimulation of OPNs (Keller et al., 2000). Electrophysiological experiments in anaesthetized cats demonstrated that stimulation of the rostral superior colliculus promoting fixation excites OPNs monosynaptically, whereas stimulation of the caudal superior colliculus promoting saccades induced monosynaptic excitation of inhibitory burst neurons (IBNs), but disynaptic inhibition of OPNs (Sugiuchi et al., 2005; Takahashi et al., 2022). Thus, premotor IBNs in the dorsal paragigantocellular nucleus do not only inhibit the contralateral abducens nucleus and excitatory burst neurons during ipsilateral horizontal saccades, but they monosynaptically inhibit the OPNs (Takahashi et al., 2022). At the same time the IBNs are tonically inhibited by OPNs during fixation as all premotor burst neurons. Based on their findings Takahashi et al., propose that premotor IBNs provide the inhibitory trigger signal required for saccade initiation, but they also induce sustained inhibition during the saccade, possibly by activation of a distinct group of early-bursting long-lead IBN found intermingled with short-lead burst neurons (Scudder et al., 1988; Cullen and Guitton, 1997; Takahashi et al., 2022).

In line with the physiological studies the glycinergic OPNs were found to receive a strong glycinergic input on their somata and proximal dendrites in monkey (Horn et al., 1994) most likely to originate from glycinergic premotor IBNs (Spencer et al., 1989; Takahashi et al., 2014). The present study revealed the expression of glycine receptor GlyR1 in human OPNs thereby supporting similar mechanism for saccade generation as found in monkey and cat.

Unlike monkey, were a strong supply of GABA- or GAD-immunoreactive terminals was found associated with the membranes of OPNs (Horn et al., 1994; Horn et al., in press), only a weak supply of GABAergic synapses was found on the somatic membranes of OPNs in human. Possible sources of GABAergic input to OPNs include monosynaptic projections from the central mesencephalic reticular formation (cMRF), where 66% of all tracer-labelled terminals associated with OPNs expressed GABA-immunolabelling (Wang et al., 2013). The cMRF has reciprocal connections with the superior colliculus and is thought to contribute to the control of primarily horizontal gaze (combined eye and head movements) via a parallel superior colliculus – cMRF – OPN pathway and/or participate in feedback pathway to the superior colliculus transmitting signals of current eye position change (Chen and May, 2000; Waitzman et al., 2002).

A further source for GABAergic input to OPNs may arise from IBNs in the interstitial nucleus of Cajal (INC) as a path for generation of vertical saccades. Only recently, Takahashi et al. demonstrated in anaesthetized cats that similar to horizontal saccadic circuitry the OPNs receive a disynaptic inhibitory input from the caudal superior colliculus via IBNs in the INC that at the same time target motoneurons in the trochlear nucleus (Takahashi et al., 2024). By combined tract-tracing in monkey a population of GABAergic premotor neurons had been identified in INC that project to motoneurons in the contralateral trochlear nucleus, which may represent IBNs of the vertical system (Horn et al., 2003b; Sugiuchi et al., 2013). A direct projection from GABAergic IBNs for vertical saccades to OPNs as seen in cat remains yet to be demonstrated in monkey. A pharmacological study in alert cats demonstrated that the iontophoretic application of the glycine receptor antagonist strychnine and the GABA-A receptor antagonist bicuculline increased the firing rate of OPNs. But the analyses of the pause timing relative to saccade onset suggest that only glycinergic afferents determine the onset and duration of pause in OPN activity during saccades in all directions, whereas blocking GABAergic input showed no effect on duration or timing of OPN pause (Kanda et al., 2007). Several studies indicate that OPNs may also play a role in the premotor circuit for other eye movement types (Mays and Gamlin, 1995; Missal and Keller, 2002; Prsa and Galiana, 2007). For example neuronal activity of OPNs is reduced by 34% during smooth pursuit and its onset coincided with the start of eye movement (Missal and Keller, 2002). Whether the reduction of OPN activity is provided by a GABAergic input, or whether GABA controls other as yet unknown aspects of OPN activity remains to be studied.

Taken together, cholinergic non-OPNs differed most clearly by their inhibitory transmitter inputs from saccadic OPNs. Unlike OPNs, the cholinergic non-OPNs lacked glycine receptors completely, but showed a strong expression for GABA receptor, accompanied by a strong supply of GAD-positive terminals. These findings indicate that the inhibitory control of cholinergic non-OPNs is provided only by GABA and not by glycine. Thereby, these neurons do not receive inhibitory inputs from neurons involved in saccade or vestibular eye movement generation, which involve glycinergic neurons (Spencer et al., 1989). Together with the cholinergic properties and the absence of perineuronal nets supporting fast firing characteristics, the lack of glycinergic input proves that the cholinergic non-OPNs represent a separate group of neurons that is different from the OPNs. On the other hand, saccadic OPNs are intermingled with cholinergic non-OPNs receiving a similarly dense and spatially distributed input from vGlut2 and vGlut1-positive terminals and therefore may share at least some sources of input with OPNs. It can be speculated that cholinergic non-OPNs may be involved in the control of different aspects of saccades or other eye movements in humans. The investigation of saccades in identical experimental setups, where a fixation target was presented in addition to a primary fixation point, revealed that monkeys showed shorter saccadic latencies and no undershooting compared to human with longer latencies and regular undershooting the visual target (Baizer and Bender, 1989). During free viewing of presented natural and artificial video clips, monkeys generated faster saccades separated by shorter fixation periods compared to humans. Further, the saccades in monkey spanned a greater range of the screen (Berg et al., 2009). However these differences were minimized, when similar high-interest saccadic targets were chosen for the experiments in both species (Berg et al., 2009). Comparative studies of smooth pursuit eye movements demonstrated higher eye velocities in monkeys with lower precision compared to humans (Churan et al., 2018). Therefore cholinergic non-OPNs present only in human may reflect additional components required for attentional and cognitive aspects of eye movements in target selection (Zhao et al., 2012).

### Limitations and considerations

Collectively, our results strongly suggest the existence of a separate neuronal population within the human RIP that could be misidentified as OPNs in anatomical and pathological investigations. For instance, when a case of saccadic palsy following cardiac surgery was subjected to post-mortem histochemical analysis, one main finding was that PNs surrounding OPNs were fragmented or completely lost (Eggers et al., 2015). Our work has demonstrated that, without the additional markers that help separate these two populations in RIP (such as ChAT and SMI32), neurons which lack PNs completely may be mistaken for OPNs with lost PNs. Indeed, in our previous investigations, we have encountered some SMI32-positive neurons with no PNs within the RIP in cases with no oculomotor disorders (but no neurons with fragmented/broken PN ensheathment) (not shown). This study provides a further clarification for the existence of such neurons.

Although a cholinergic non-OPN population within human RIP was identified and characterized by their ion channel and transmitter profiles to deduce their biophysiological features, our methodology does not allow us to further suggest a functional role to these neurons. Lack of this cholinergic population in RIP of macaque monkey also hinders possible tract-tracing experiments to reveal their connectivity. Therefore,it is unlikely that electrophysiological recordings characterizing OPNs encountered such non-OPNs mistakenly in monkey. The expression of SMI32 immunoreactivity implies that these cholinergic non-OPNs in human could be projection neurons, with possibly smaller capacity for high rate of fire than OPNs as indicated by the lack of PN and less Kv3.1b expression. It might be worthwhile to assess the existence of such a population in other animals such as cats and mice which allows for a wider variety of methods to confirm the connectivity of these neurons.

Peroxidase-based immunohistochemistry method does not allow quantitative evaluation of ion channel expression, since staining quality varies depending on multiple factors associated with processing human tissue, such as post-mortem delay and paraformaldehyde fixation duration. However with this method fundamental differences about protein expression related to transmitters can be detected especially when combined for pre- and postsynaptic markers and shown here for GABA.

## Data Availability

The raw data supporting the conclusions of this article will be made available by the authors, without undue reservation.
